# Role of CD40 ligand in transfusion-related acute lung injury

**DOI:** 10.1186/cc9844

**Published:** 2011-03-11

**Authors:** PR Tuinman, M Gerards, G Jongsma, L Boon, AP Vlaar, NP Juffermans

**Affiliations:** 1 Academic Medical Center, Amsterdam, the Netherlands; 2 Bioceros, Utrecht, the Netherlands

## Introduction

Transfusion-related acute lung injury (TRALI) is an important problem in the critically ill. CD40L has been implicated as a cofactor or even the cause of TRALI [1]. (1) We hypothesized that blocking of the CD40-CD40L interaction protects against lung injury in a murine model of TRALI. (2) Furthermore, we hypothesized that plasma sCD40 levels are elevated in TRALI patients compared with controls.

## Methods

(1) Male BALB/c mice (*n *= 96) were challenged with monoclonal MHC-1 antibody (Ab), a previously used murine TRALI model [2]. In separate experiments, mice were pretreated with Ciglitazone, an inhibitor of the expression of CD40L on platelets, or anti-CD40L Ab, which antagonizes CD40L-CD40 interaction. Controls received vehicle or isotype Ab. After 2 hours, mice were killed and bronchoalveolar lavage fluid (BALF) was obtained. The wet lung to body weight ratio was calculated. Total protein, keratinocyte-derived chemokine (KC) and macrophage-inflammatory protein-2 (MIP-2) were measured in BALF. (2) Cardiac surgery patients were prospectively followed for the onset of TRALI (by applying the consensus definition). Sixteen TRALI cases were compared with controls (transfused patients not developing lung injury). Plasma levels of sCD40L were measured before surgery and at onset of TRALI.

## Results

(1) Infusion of MHC-1 Ab resulted in pulmonary edema, accompanied by elevated BALF levels of total protein and KC and MIP-2 compared with infusion of isotype Ab (*P *< 0.05 to all). Treatment with ciglitazone or anti-CD40L Ab did not result in a decrease of pulmonary edema compared with MHC-1 Ab, nor did it reduce BALF KC levels (33 ± 6.1 vs. 27 ± 19 vs. 13 ± 9.4 ng/ml respectively) and MIP-2 levels (5.3 ± 3.0 vs. 1.5 ± 1.7 vs. 2.0 ± 2.9 ng/ml respectively). (2) Surgery resulted in a decrease in sCD40L levels, with a concomitant decrease in platelet count. We found no difference in plasma levels of sCD40L between cardiac surgery patients developing TRALI and controls (275 ± 192 vs. 258 ± 346 and 93 ± 82 vs. 93 ± 123 pg/ml respectively, NS).

## Conclusions

CD40L does not play a role in this model of antibody-mediated TRALI. Moreover, sCD40L levels are not different in cardiac surgery patients developing TRALI compared with transfused controls, further suggesting that sCD40L is not a mediator of TRALI.
